# Significance of Multiple Bioactivation Pathways for Meclofenamate as Revealed through Modeling and Reaction Kinetics[Fn FN3]

**DOI:** 10.1124/dmd.120.000254

**Published:** 2021-02

**Authors:** Mary Alexandra Schleiff, Noah R. Flynn, Sasin Payakachat, Benjamin Mark Schleiff, Anna O. Pinson, Dennis W. Province, S. Joshua Swamidass, Gunnar Boysen, Grover P. Miller

**Affiliations:** Departments of Biochemistry and Molecular Biology (M.A.S, G.P.M.) and Environmental and Occupational Health (G.B.), University of Arkansas for Medical Sciences, Little Rock, Arizona (M.A.S.); Department of Pathology and Immunology, Washington University, St. Louis, Missouri (N.R.F., S.J.S.); Department of Chemistry, Hendrix College, Conway, Arizona (S.P.); and Independent Researcher (B.M.S.) and Department of Chemistry and Biochemistry (A.O.P., D.W.P.), Harding University, Searcy, Arkansas

## Abstract

Meclofenamate is a nonsteroidal anti-inflammatory drug used in the treatment of mild-to-moderate pain yet poses a rare risk of hepatotoxicity through an unknown mechanism. Nonsteroidal anti-inflammatory drug (NSAID) bioactivation is a common molecular initiating event for hepatotoxicity. Thus, we hypothesized a similar mechanism for meclofenamate and leveraged computational and experimental approaches to identify and characterize its bioactivation. Analyses employing our XenoNet model indicated possible pathways to meclofenamate bioactivation into 19 reactive metabolites subsequently trapped into glutathione adducts. We describe the first reported bioactivation kinetics for meclofenamate and relative importance of those pathways using human liver microsomes. The findings validated only four of the many bioactivation pathways predicted by modeling. For experimental studies, dansyl glutathione was a critical trap for reactive quinone metabolites and provided a way to characterize adduct structures by mass spectrometry and quantitate yields during reactions. Of the four quinone adducts, we were able to characterize structures for three of them. Based on kinetics, the most efficient bioactivation pathway led to the monohydroxy para-quinone-imine followed by the dechloro-ortho-quinone-imine. Two very inefficient pathways led to the dihydroxy ortho-quinone and a likely multiply adducted quinone. When taken together, bioactivation pathways for meclofenamate accounted for approximately 13% of total metabolism. In sum, XenoNet facilitated prediction of reactive metabolite structures, whereas quantitative experimental studies provided a tractable approach to validate actual bioactivation pathways for meclofenamate. Our results provide a foundation for assessing reactive metabolite load more accurately for future comparative studies with other NSAIDs and drugs in general.

**Significance Statement:**

Meclofenamate bioactivation may initiate hepatotoxicity, yet common risk assessment approaches are often cumbersome and inefficient and yield qualitative insights that do not scale relative bioactivation risks. We developed and applied innovative computational modeling and quantitative kinetics to identify and validate meclofenamate bioactivation pathways and relevance as a function of time and concentration. This strategy yielded novel insights on meclofenamate bioactivation and provides a tractable approach to more accurately and efficiently assess other drug bioactivations and correlate risks to toxicological outcomes.

## Graphical abstract

**Figure:**
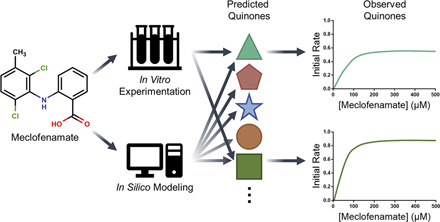

## Introduction

Nonsteroidal anti-inflammatory drugs (NSAIDs) are effective in the treatment of rheumatoid arthritis and osteoarthritis, dysmenorrhea, menorrhagia, and fever (McLean and Gluckman, 1983; Conroy et al., 1991; Niazy, 1996; Wolfe et al., 1999), yet they often carry a significant risk of adverse drug events (Wolfe et al., 1999; Galati et al., 2002; Goldkind and Laine, 2006; Hawboldt, 2008; Li et al., 2009; Davis and Robson, 2016). In the 1960s and 1970s, attempts to synthesize new NSAIDs with reduced gastrointestinal toxicity led to the development of the fenamate class of NSAIDs comprising meclofenamate, flufenamate, mefenamate, and tolfenamate (Wolfe et al., 1999). However, this new class of drugs carried risks for inducing hepatic and bone marrow toxicities, resulting in decreased but persistent use in the clinic (Galati et al., 2002; Somchit et al., 2004; Goldkind and Laine, 2006; Narsinghani and Chaturvedi, 2006; Aronson, 2016). Unlike with NSAID-induced gastrointestinal effects, few studies have been conducted to identify the mechanism for fenamate hepatotoxicity (Reinicke and Klinger, 1971; Wolfe et al., 1999; Galati et al., 2002; Somchit et al., 2004; Sriuttha et al., 2018). The proposed cause for this toxicity may arise from cytochromes P450 bioactivation of fenamates into reactive quinones that form adducts with hepatic proteins and glutathione (Galati et al., 2002). Nevertheless, the possible bioactivation pathways that exist and their relevance to overall metabolism of fenamates remain unknown. This knowledge would provide important mechanistic information for more accurate risk assessments for patients taking NSAIDs.

Traditional bioactivation assessments for drugs involve reactions with human liver microsomes in the presence of reagents, such as cyanide or glutathione, which trap reactive metabolites (Stachulski et al., 2013). The resulting stable adducts are frequently detected by mass spectrometry to flag problematic molecules (Evans et al., 2004; Argoti et al., 2005; Ma and Zhu, 2009). Nevertheless, the high dependence of mass-spectroscopic response on structure and the frequent absence of authentic standards make these assessments qualitative and not quantitative, so it is not possible to scale the extent and hence relevance of bioactivation (Dahal et al., 2011; Hatsis et al., 2017; Pinson et al., 2020). The gold standard for quantitation of reactive metabolite adducts relies on low throughput and costly radiolabeling experiments (Zhou, 2003; Evans et al., 2004). A critical advance in the field was the development and validation of dansyl glutathione as a more economical reagent to trap, track, and quantitate reactive quinone adducts (Gan et al., 2005). Its application toward a series of drugs demonstrated the expected positive trend toward adduct formation and drug-induced liver injury when coupled to bioavailability and dose to yield a daily dose burden of reactive metabolites (Gan et al., 2009). Nevertheless, a limitation of this study was the measurement of a bioactivation rate at a single drug concentration, as is common in these types of studies. In practice, drug exposure levels vary, but the relationship between bioactivation rates and drug concentrations provides the capacity to scale and compare relative bioactivation risks.

As an alternative, steady-state studies characterize reaction mechanisms describing drug binding (K_m_), maximal metabolic rate (V_max_), and catalytic efficiency (V_max_/K_m_). Those kinetic constants then provide a basis for assessing the relative importance of individual and competing metabolic pathways for individual drugs and arrays of drugs. For bioactivation, we previously applied this kinetic approach to assess N-dealkylation of the antifungal drug terbinafine involving formation of a reactive allylic aldehyde trapped by dansyl hydrazine (Barnette et al., 2018). Steady-state kinetics for reactions revealed the preferred bioactivation pathway among three possibilities. In follow-up studies, we determined the relative significance of cytochrome P450 isozymes contributing to terbinafine bioactivation pathways and extrapolated their relevance for the average adult (Barnette et al., 2019; Davis et al., 2019). Similarly, we studied bioactivation kinetics for a pair of NSAIDs, sudoxicam and meloxicam, to yield a thioamide protoxin and dicarbonyl cometabolite (Barnette et al., 2020). Based on metabolic kinetics, the sole methyl group difference among the drugs resulted in decreased bioactivation and increased detoxification for meloxicam. This observation may explain the much-lower toxicity reported for meloxicam over sudoxicam, the latter of which was discontinued because of high incidences of hepatoxicity in clinical trials. In following, steady-state kinetics provide a powerful strategy for assessing the potential relevance of drug bioactivations.

For this study, we are the first to measure bioactivation kinetics for meclofenamate into reactive quinone-species metabolites through a novel application of the dansyl glutathione trap. As a first step, we used computational models developed by our group to obtain a rapid snapshot of the likelihood for possible quinone reactions and resulting reactive metabolite structures (Hughes et al., 2015, 2016; Hughes and Swamidass, 2017; Flynn et al., 2020). For experimental kinetics studies, accurate quantitation of adducts required that structural variations among them did not impact fluorescence of the adducting molecule, dansyl glutathione. We validated this assumption with a structurally diverse set of dansylated molecules. After this verification, we carried out metabolic reactions with meclofenamate using pooled human liver microsomes in the presence of the dansyl glutathione trap for reactive quinone-species metabolites. We resolved the resulting adducts chromatographically, characterized structures by mass spectrometry, and quantitated yields based on dansyl fluorescence. After optimizing reaction conditions, we determined steady-state kinetics leading to multiple quinone-species metabolites for meclofenamate. The significance of those pathways would ultimately depend on contributions relative to overall meclofenamate metabolism, and thus we measured steady-state kinetics for parent-drug depletion and calculated corresponding fractional bioactivations.

## Methods

### 

#### Materials.

All chemical solvents were purchased from Thermo Fisher Scientific (Waltham, MA). The following chemicals were purchased from Millipore-Sigma (Burlington, MA): substrate meclofenamic acid (meclofenamate); internal standard dansylamide; reducing agent Tris(2-carboxyethyl)phosphine hydrochloride; NADPH-regenerating system components NADP disodium salt, glucose-6-phosphate dehydrogenase, and glucose-6-phosphate, as well as dansyl cadaverine and dansyl amidoethylmercaptan. Magnesium chloride salt was purchased from Thermo Fisher Scientific. Trapping agent dansyl glutathione trifluoroacetic acid salt was purchased from Toronto Research Chemicals (Toronto, ON, Canada). Human liver microsomes pooled from 150 donors [human liver microsomes 150 (HLM150)] were purchased from Corning Gentest (Woburn, MA). Marvin 20.4 was used for drawing, displaying, and characterizing chemical structures, substructures, and reactions (http://www.chemaxon.com; ChemAxon).

#### Predicting Meclofenamate Bioactivation into Quinone-Species Metabolites and Subsequent Reactivity.

As an initial analysis, we rapidly predicted bioactivation pathways for meclofenamate by coupling a series of models. First, we modeled quinone formation to reveal potential "hot spot" sites involved in bioactivation of the drug (Hughes and Swamidass, 2017). This deep neural network model predicts one- and two-step quinone formation by identifying atom pairs at which metabolic oxidation may occur to form quinone metabolites with an accuracy of 88.2% as determined via receiver operating characteristic curve analyses. Second, we used these model outputs to predict structures for quinone metabolites using the XenoNet model and scaled their likelihood based on the quinone model predictions (Flynn et al., 2020). This model functions via the input of a substrate and optional target product pair and enumerates pathways of intermediate metabolite structures while computing likelihood scores for each pathway. Third, we modeled the reactivity of the individual quinone metabolites toward glutathione as a trap (Hughes et al., 2015, 2016). This model predicts sites and likelihood for reactivity of inputted molecules with major biomolecules, such as glutathione and proteins. Each model scales scores differently, and thus we relied on the quinone model values as the final arbiter of possible bioactivation potential for meclofenamate.

#### Steady-State Kinetics for Meclofenamate Quinones Trapped with Dansyl Glutathione.

The in vitro meclofenamate studies relied on reactions with HLM150 as a model for the average adult human liver. Microplate half-wells containing HLM150, 1 mM dansyl glutathione, and substrate in 100 mM potassium phosphate buffer, pH 7.4, with 0.1% DMSO as cosolvent were preincubated for 5 minutes at 37°C with shaking at 350 rpm using a BMG Labtech THERMOstar incubator (Ortenberg, Germany). Control studies were initially carried out to identify suitable steady-state conditions for the final HLM150 protein concentrations and reaction times. Specific substrate concentrations were varied from 0 to 500 µM, biasing specific values to better reveal the relationship between initial rates and substrate concentration. Reactions were initiated upon addition of an NADPH-regenerating system (0.4 units/μl glucose-6-phosphate dehydrogenase, 10 mM glucose 6-phosphate, 2 mM MgCl_2_, 500 μM NADP+). Identical mixtures without addition of NADPH-regenerating system were incubated as negative controls. Aliquots were quenched by adding 2-fold volume of ice-cold methanol containing an internal standard (10 μM dansylamide) and reducing agent (5 mM Tris(2-carboxyethyl)phosphine) (Amaya et al., 2018). The total mixture was chilled on ice for 10 minutes to optimize precipitation of proteins and phosphate buffer (Schellinger and Carr, 2004). After 2800*g* centrifugation at 4°C for 15 minutes using a Sorvall ST 16R Centrifuge (Thermo Scientific), the supernatant was transferred to a 96-well full-volume microplate and evaporated to dryness using an Organomation Microvap Nitrogen Evaporator System (Organomation Associates, Inc, Berlin, MA). Dried wells were then resuspended in mobile phase (20:80 water:acetonitrile + 0.1% formic acid) for high-pressure liquid chromatography coupled with UV-visible/fluorescence detection and analyzed as described in the following section. Each set of steady-state reactions was performed in triplicate and replicated three times. Initial rates were calculated and plotted against substrate concentration and then fit to the Michaelis-Menten equation, the summation of two Michaelis-Menten equations, or the Hill positive cooperativity model using GraphPad Prism 7.0 from GraphPad Software, Inc (San Diego, CA). The best-fit kinetics model and corresponding constants were determined using the extra sum-of-squares F test. In addition, we excluded kinetic mechanisms, which had open confidence intervals for best-fit values.

#### Analysis of Meclofenamate Bioactivation Reactions.

Sample reactions were analyzed to quantitate reactive metabolite adducts by fluorescence, and then their structures were characterized by mass spectrometry. Reaction metabolites were separated by a 4.6 × 150 mm Waters XSelect HSS C18 3.5-µm column using a Shimadzu LC-20AB Prominence liquid chromatograph and detected by a Shimadzu RF-10AXL fluorescence detector or a Shimadzu SPD-10A VP UV-visible detector. Mobile phase consisted of solvents A (0.1% formic acid in 90:10 deionized water:acetonitrile) and B (0.1% formic acid in acetonitrile). The gradient method started with 89% solvent A and decreased to 67% over 3 minutes, then decreased to 56% A over 9 minutes, and then decreased again to 33% A over 5 minutes. Solvent A was increased back to 89% over 3 minutes and held for remainder of run. The total flow rate was 1 ml/min, and total run time per sample was 25 minutes. The fluorescence detector was set to emit an excitation energy of 340 nm and detect an emission energy of 525 nm to optimally detect dansyl fluorescence (Gan et al., 2005, 2009; Amaya et al., 2018). The absorbance detector was set to detect an absorbance of 270 nm for simultaneous detection of substrate depletion in conjunction with substrate bioactivation (Gouda et al., 2013). Substrate standard curves began to plateau at higher concentrations, and thus data were fitted to a quadratic equation rather than linear regression to avoid underestimation of substrate depletion at higher concentrations. Analyte responses were normalized to internal standard dansylamide and quantitated relative to fluorescent response of a dansyl glutathione standard dilution. Control studies were conducted to ensure that dansyl fluorescence did not depend on the specific adduct structure, thus obviating the need for adduct standards to quantitate fluorescent responses. Resultant values were used to calculate initial reaction rates for both parent-drug depletion and bioactivation.

Although quantitative, fluorescence response provides no structural information for adducts, and thus we analyzed dansyl glutathione–adducted metabolites by mass spectrometry to determine parent masses and fragmentation patterns for inferring purported adduct and reactive metabolite structures. Samples were injected onto an Agilent Technologic 1290 Infinity HPLC using the same chromatographic method and column as described previously. Analytes were characterized with the Agilent Technologic 6490 Triple Quad LC/MS. The electrospray ionization source was operated in negative and positive ion mode, and ion spectra were acquired in full-scan mode monitoring the mass-to-charge ratio range of 100–1200 amu. Subsequently, product ion spectra were generated from precursor ions with monitoring for fragmentation by collision-induced dissociation (30 eV) with a range of 45–1000 amu in negative ion mode.

## Results

### 

#### Modeling Predicted Meclofenamate Bioactivation through Diverse Pathways Varying in Likelihood.

**Fig. 1.**
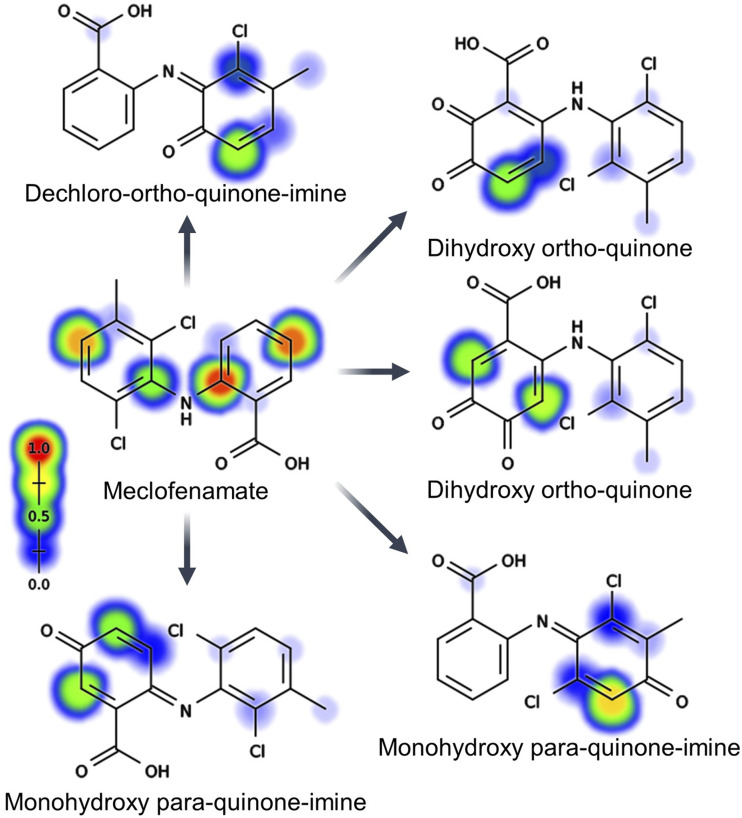
Prediction of bioactivation of meclofenamate and reactivity of selected metabolites with glutathione. Computational model outputs are shown for the parent drug, meclofenamate, and a sampling of the predicted reactive quinone metabolites. The model used for the parent drug predicts molecular-level and atom-level likelihood for bioactivation of the compound into a quinone-species metabolite (Hughes and Swamidass, 2017). In total, 19 predicted metabolite structures were then automatically generated (Flynn et al., 2020). The model used for the computationally generated metabolites predicts molecular-level and atom-level likelihood of glutathione reactivity (Hughes et al., 2015, 2016). Atom-by-atom scores are shown visually by red (1.0) to light blue (0.0), with high (red, 1.0) scores indicating a high likelihood for hydroxylation and subsequent bioactivation into quinones and low (light blue, 0.0) scores indicating a low likelihood for hydroxylation and subsequent bioactivation into quinones. Both numerical and graphical data for all predicted metabolites can be accessed in Supplemental Files 1–3.

Modeling of meclofenamate indicated a high likelihood for bioactivation into a reactive quinone-species metabolite from an overall model score of 0.93 (Fig. 1; Supplemental Information). Subsequent modeling of the predicted corresponding quinone-species metabolites identified both the 2,4-dichloromethylbenzene and the benzoic acid functional groups as bioactivation targets. Predicted quinone-species metabolite structures included chlorinated and dechlorinated monohydroxy para- and ortho-quinone-imines, monohydroxy ortho-quinone-methides, dihydroxy ortho-quinones, and dechlorinated ortho-quinones and ortho-quinone-methides (Fig. 1; Supplemental Information). The most highly predicted metabolites were monohydroxy para-quinone-imines on both the acidic aromatic ring and the chlorinated aromatic ring, with the former being more favorable. Exceptions for the trend involved metabolic dechlorination followed by subsequent metabolic oxidation reactions of the 2,4-dichloromethylbenzene. The potential reactivities for these metabolites toward critical biomolecules, such as glutathione and proteins, are shown in Fig. 1 and Supplemental Information. They are expectedly highly predicted, with quinone-methide–containing metabolites being the most reactive. Full numerical and graphical results for these computational outputs are contained within the Supplemental information.

#### Mass-Spectroscopic Studies Characterize Fluorescent Dansyl Glutathione Adducts.

We assessed predicted bioactivations using mass-spectroscopic fragmentation approaches to characterize structures of quinone adducts trapped during meclofenamate microsomal reactions. Meclofenamate possesses an asymmetric diphenylamine motif that undergoes bioactivation into ortho- or para-quinones with a potential for dehalogenation. Parent and product ion scans were performed to identify putative adduct parent masses as well as characteristic dansyl glutathione fragments. Four fluorescent adducts were observed after meclofenamate metabolism. Several species of reactive quinone metabolites were characterized from mass-spectroscopic fragmentation: dihydroxy ortho-quinones, monohydroxy para-quinone-imines, and dechlorinated ortho-quinone-imines (Supplemental Fig. 5). Shown in Fig. 3 are chromatograms, fragmentation spectra, and adduct structures for the meclofenamate monohydroxy para-quinone-imine (mass-to-charge ratio 848.1). Fragments 234, 252, 361, 378, 487, 505, and 539 are characteristic dansyl glutathione fragments previously reported by Gan and colleagues (2005, 2009), whereas other fragments were predicted using Competitive Fragmentation Modeling for Metabolite Identification 3.0 (Allen et al., 2014, 2015; Djoumbou-Feunang et al., 2019). For each labeled metabolite, at least two characteristic dansyl glutathione fragments were detected, with most metabolites producing most or all the previously reported fragments. Additionally, parent masses for meclofenamate and monohydroxylated metabolites were observed, although hydroquinone precursors could not be detected because of their high reactivity under reaction conditions (Supplemental Fig. 1).

#### Control Studies Established Reliability of Quantitative Kinetics for Meclofenamate Bioactivations.

**Fig. 2.**
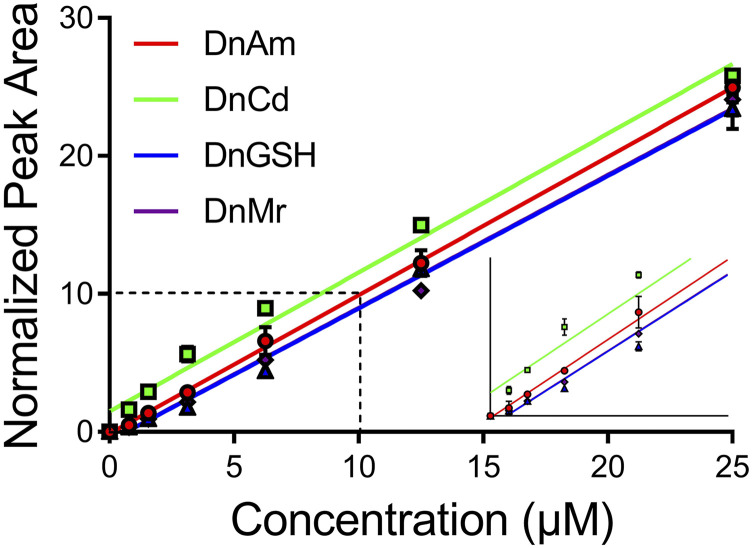
Dansyl fluorescence is independent of remainder of molecule structure. Standard curves from 0 to 25 µM for four dansylated compounds—specifically DnAm, dansylamide; DnCd, dansyl cadaverine; DnGSH, dansyl glutathione; and DnMr, dansyl amidoethylmercaptan—were measured for fluorescent response and fitted with simple linear regressions to determine whether dansyl fluorescent response was independent of attached moieties and thus facilitating quantification of dansyl-adducted metabolites. All fluorescent responses were normalized to dansylamide fluorescent responses to highlight the similar absolute fluorescence intensity and relationship to concentration for all dansylated derivatives. The figure inset displays truncated *x*- and *y*-axes to show the lower concentration points in better detail.

For kinetic studies, we determined steady-state conditions for measuring accurate initial rates and validated the utility of the dansyl label for quantitation of meclofenamate adducts. First, we chromatographically resolved quinone-species metabolite adducts from reactions based on fluorescence and identified linearity as a function of time (Supplemental Fig. 2) and protein concentration (Supplemental Fig. 3). Higher-yield adducts formed linearly up to 60 minutes and 1 mg/ml protein concentration, whereas linearity was observed for lower-yield adducts only up to 0.25 mg/ml protein. From these results, we selected a 60-minute reaction time and 0.25 mg/ml protein for steady-state experiments. Second, the quantitation of adducted reactive metabolites depends on the independence of dansyl fluorescence from the remainder of the molecule structure. As a test, dansylamide, dansyl cadaverine, dansyl glutathione, and dansyl amidoethylmercaptan were shown to have similar fluorescence responses after normalization to dansylamide responses with slopes ranging from 0.96 for dansyl glutathione to 1.01 for dansyl cadaverine (variability < 5%) and with R^2^ values for quality of linear fits ranging from 0.99 for dansyl cadaverine to 1.0 for dansylamide as a function of concentration up to 25 µM (Fig. 2). Thus, structural differences among reactive metabolite adducts would not likely impact dansyl fluorescence, making their quantitation possible in the absence of authentic standards for kinetic studies.

#### Steady-State Kinetics Revealed Major and Minor Bioactivation Pathways for Meclofenamate.

Characterization of adduct structures by mass spectrometry provided a strategy for correlating specific bioactivation pathways to quantify reaction kinetics using fluorescent responses from labeled, trapped meclofenamate quinones. A critical first step was the chromatographic resolution of these reactive metabolites as shown in the fluorescence chromatogram for meclofenamate metabolism (Fig. 4). Quantitation of fluorescence response as a function of time and concentration resulted in kinetic profiles that are shown in Fig. 5. Metabolism of meclofenamate into the dechloro-ortho-quinone-imine demonstrated slight positive cooperativity, and using the Hill equation yielded the highest rate of turnover (V_max_) and a moderate Hill constant (K_h_) for the midpoint of the curve. Kinetics for the monohydroxy para-quinone-imine conformed to the Michaelis-Menten mechanism with the second highest V_max_ and the lowest K_m_. The similar dihydroxy ortho-quinone showed significant positive cooperativity with a moderate V_max_ and weak meclofenamate binding based on its K_h_. The likely multi-GSH adduct involved Michaelis-Menten kinetics for the least-efficient bioactivation pathway because of the lowest V_max_ and a moderate K_m_. These data were then used to estimate fractional bioactivation contributions and estimate load burdens from the reactive metabolites.

**Fig. 3.**
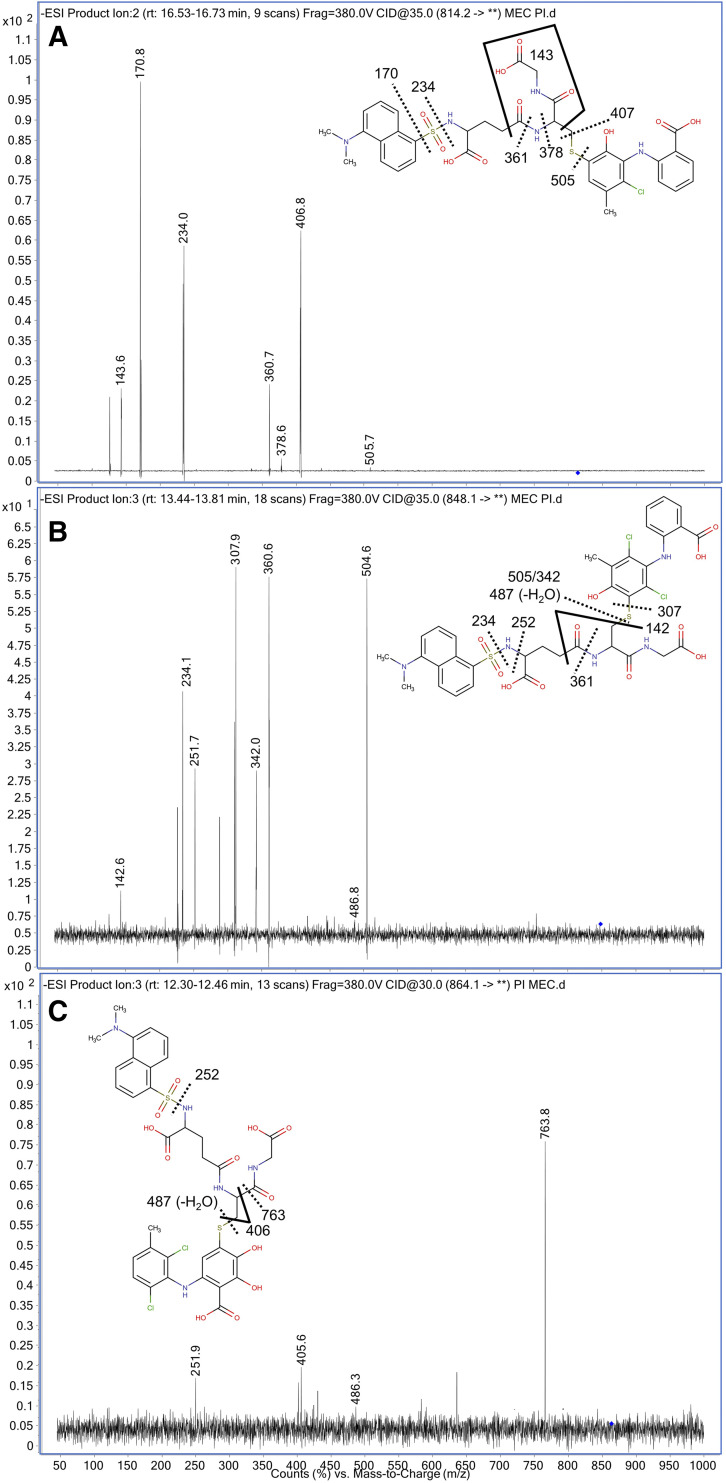
Mass-spectroscopic product ion spectra and adduct structures for meclofenamate metabolites dechloro-ortho-quinone-imine (A), monohydroxy para-quinone-imine (B), and dihydroxy ortho-quinone (C).

**Fig. 4.**
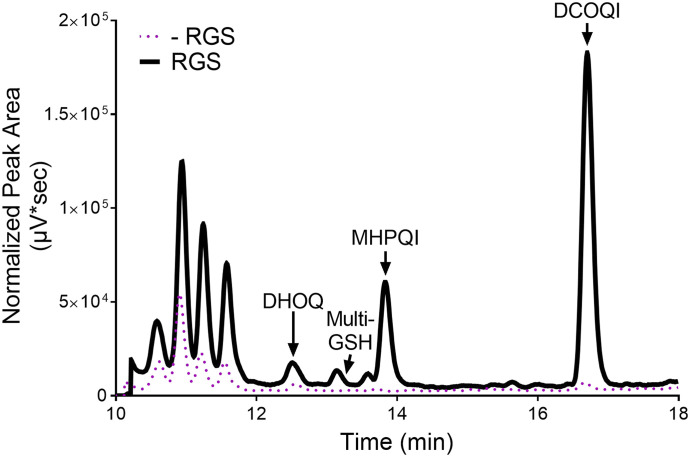
Fluorescent chromatogram of 500 µM meclofenamate metabolism over 60 minutes by 1 mg/ml human liver microsomes with 1 mM dansyl glutathione. Two experimental conditions are shown: reactions with NADPH [black, NADPH-regenerating system (RGS)] and reactions without NADPH (pink, - RGS), which serves as a negative control. Unique peaks denote dansyl glutathione–adducted metabolites. Structures of these metabolites were characterized via product ion mass spectrometry and are labeled as follows: DCOQI, dechloro-ortho-quinone-imine; DHOQ, dihydroxy ortho-quinone; MHPQI, monohydroxy para-quinone-imine; and Multi-GSH, a suspected multiply glutathionylated metabolite. Chromatograms were adapted using GraphPad Prism 7.0 from Shimadzu LCSolution to improve visibility.

**Fig. 5.**
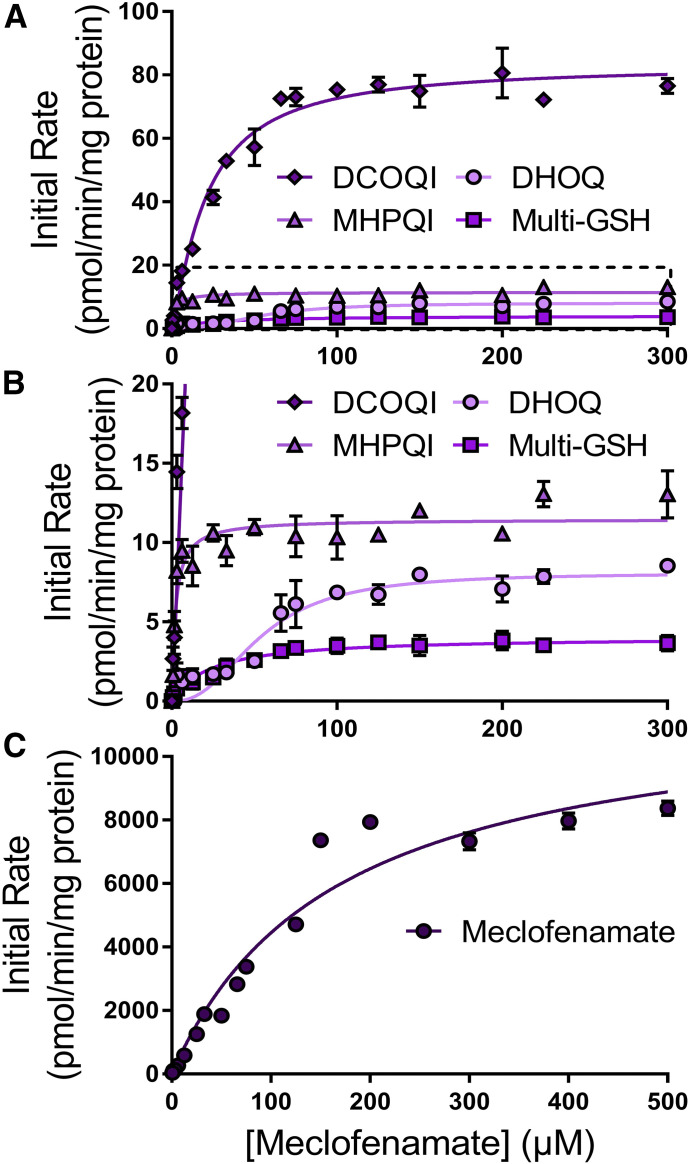
Steady-state kinetics for meclofenamate metabolism. Reaction conditions and data analysis were carried out as described in *Methods*. Each point is the avg. of three to six replicates. (A) shows kinetic profiles for dansyl glutathione–adducted metabolites generated during meclofenamate metabolism, with corresponding constants reported in Table 1. Although data were collected and fit to meclofenamate concentrations up to 500 µM, both (A and B) are truncated to 300 µM to highlight the shapes of the curves. Alternative figures with concentrations up to 500 µM are shown in Supplemental Fig. 4. (B) is a version of the data shown in (A) with a truncated *y*-axis to highlight the fit and trends of the three less-efficient metabolite formation rates. (C) shows the kinetic profile for meclofenamate depletion reflecting overall metabolic clearance of the drug with corresponding constants reported in Table 2. Abbreviations are as follows: DCOQI, dechloro-ortho-quinone-imine; DHOQ, dihydroxy ortho-quinone; MHPQI, monohydroxy para-quinone-imine; and Multi-GSH, a suspected multiply glutathionylated metabolite.

#### Kinetics Revealed Individual and Combined Relative Burden of Meclofenamate Quinones.

The exposure load of reactive metabolites arises from the efficiency of bioactivation relative to overall metabolism of the drug. We assessed this fractional bioactivation (f_ba_) by calculating ratios of catalytic efficiencies for meclofenamate bioactivation and depletion for individual pathways and a combination of all possibilities (Table 1). The kinetics for monohydroxy para-quinone-imine and multiply glutathionylated adduct conformed to the Michaelis-Menten mechanism so that metabolic efficiency corresponded to V_max_/K_m_. In contrast, the dechloro-ortho-quinone-imine and dihydroxy ortho-quinone formation demonstrated positive cooperativity. Consequently, we approximated efficiency using the V_max_ divided by K_h_, which may slightly overpredict the efficiency at low meclofenamate concentrations. Based on this analysis, the fractional bioactivation percentages for the adducted metabolites, dechlorinated ortho-quinone-imine, monohydroxy para-quinone-imine, dihydroxy ortho-quinone, and suspected multiply glutathionylated metabolite were 3.8%, 5.0%, 0.2%, and 0.3%, respectively (Table 1). When combined, the total fractional bioactivation of meclofenamate was 0.128, or 12.8%.

**TABLE 1:** Steady-state kinetics for meclofenamate metabolism Shown are kinetic constants for the metabolism of the substrates in bold. Best-fit constants reported with 95% confidence intervals shown in parentheses.

Analyte	V_max_*^a^*	K_m_ or K_h_ (μM)	h	V_max_*^b^*/K_m_ or K_h_	Mechanism*^c^*
Meclofenamate	11,800 (10,700–13,200)	166 (133–210)		**71.1** (51.0–99.2)	Michaelis-Menten
Dechloro-ortho-quinone-imine	83.2 (80.1–87.0)	22.0 (18.8–25.7)	1.2 (1.0–1.4)	**3.8***^d^* (3.1–4.6)	Positive cooperativity
Monohydroxy para-quinone-imine	11.5 (11.0–12.0)	2.3 (1.6–3.2)		**5.0** (3.4–7.5)	Michaelis-Menten
Dihydroxy ortho-quinone	8.1 (7.5–9.0)	67.4 (47.8–93.6)	2.4 (1.5–4.0)	**0.12***^d^* (0.08–0.19)	Positive cooperativity
Multi-GSH adduct	4.1 (3.8–4.4)	23.2 (16.5–32.0)		**0.18** (0.12–0.27)	Michaelis-Menten

^*a*^ pmol/min per milligram protein.

^*b*^ pmol/min per milligram protein per micromolar.

^*c*^ Most statistically favored kinetic mechanisms are listed.

^*d*^ For positive cooperativity, catalytic efficiency is poor at low substrate concentration and improves at higher concentration so that there is not a single catalytic efficiency for the reaction. Nevertheless, fractional bioactivation analyses (Table 2) relied on V_max_/K_h_ ratio as an upper limit on a linearly dependent catalytic efficiency for bioactivation as a function of meclofenamate concentration.

## Discussion

### 

#### Combined Modeling Identified Possible Bioactivations and Supported Experimental Validations.

Computational modeling is frequently used in drug development processes to identify problems in lead compound druggability (Schleiff et al., 2020). Such models yield readily accessible data on bioactivation potential for molecules of interest without significant investments in time, effort, or resources. In this study, we combined models of bioactivation, metabolite structure, and reactivity to construct possible bioactivation pathways for meclofenamate. The predicted bioactivation of meclofenamate into a quinone was high (0.93, scale from 0.0 to 1.0), with multiple “hot spots” suggesting different possible bioactivations varying in likelihood of occurrence. To our knowledge, no previous studies identified these potential mechanisms or sites of meclofenamate bioactivation. Furthermore, we generated structures for 19 reactive metabolites (Supplemental Figs. 7 and 8) and used reactive likelihood scores to scale subsequent formation of glutathione adducts. Despite 19 possibilities, only four adducts were experimentally observable reflecting possible experimental limitations and/or poor predictions. The inability to differentiate between true and false leads for modeled bioactivations remains a challenge; however, rapid generation of metabolite structures and scaling their formations yielded useful information to guide analysis of complex experimental data to validate bioactivation pathways.

We compared measured catalytic efficiencies with scale and compared observed and predicted bioactivations. Overall, computational bioactivation predictions were in fair agreement with experimental catalytic efficiencies and fractional bioactivation values for the four unique quinone-species metabolites observed after meclofenamate metabolism (Table 2). The monohydroxy quinone-imines were the most highly predicted metabolites and most catalytically efficient reactions. Similarly, less-efficient reactions leading to dechlorinated monohydroxy quinone-imine metabolites and dihydroxy quinones corresponded to lower computational likelihood scores.

**TABLE 2:** Fractional bioactivation and computational scores for each observed meclofenamate metabolite and corresponding adduct

Analyte	Model Score*^a^*	Depletion V_max_/K_m_	Bioactivation V_max_/K_m_ or K_h_	F_ba_*^b^*	Bioactivation (%)
Dechloro-ortho-quinone-imine	0.26		3.8 (3.1–4.6)	0.053 (0.031–0.090)	5.3 (3.1–9.0)
Monohydroxy para-quinone-imine	0.67/0.92*^c^*		5.0 (3.4–7.5)	0.070 (0.034–0.147)	7.0****(3.4–14.7)
Dihydroxy ortho-quinone	0.42/0.45*^c^*		0.12 (0.08–0.19)	0.002 (0.001–0.004)	0.2 (0.1–0.4)
Multi-GSH adduct	NP*^d^*		0.18 (0.12–0.27)	0.003 (0.001–0.005)	0.3 (0.1–0.5)
Total		71.1 (51.0–99.2)	9.1 (6.7–12.6)	0.128 (0.067–0.246)	12.8 (6.7–24.6)

^*a*^ Model scores reflect the relative likelihood for metabolite formation after metabolism of the parent drug, meclofenamate. Scores range from 0.0 to 1.0, with higher scores indicating a greater likelihood for formation of the specific metabolite structure.

^*b*^ Fractional bioactivation (F_ba_) is defined as a fraction of metabolic bioactivation catalytic efficiency and metabolic depletion catalytic efficiency.

^*c*^ Multiple bioactivation scores reflect different possible reactive metabolite isomers whose structures could not be definitively attributed to the observed metabolite based on mass spectrometry.

^*d*^ No prediction (NP) for putative metabolite and adduct because of the inability to characterize its structure by mass spectrometry.

Despite general consistency, there were differences between computationally predicted and experimentally observed bioactivations. Modeling results reflected the ranking of dihydroxy quinone metabolites as more likely than formation of dechlorinated monohydroxy quinone-imine metabolites, which did not hold true experimentally. Differences in rankings and prediction of unobservable quinones may reflect the impact of limited training set size and molecular diversity on model inference of bioactivations. Model predictions do not incorporate the impact of time and concentration on metabolism observed through experimental studies, with both parameters directly impacting the identity and relative abundance of metabolites. Moreover, the training set encompassed reactions reported from several model systems ranging from in vitro systems (human liver microsomes and hepatocytes) to more complex in vivo systems (rodents, humans, and others). This varied combination of data types may not accurately reflect what is experimentally possible with human liver microsomal reactions specifically resulting in overprediction or underprediction of putative metabolites. Knowledge of these possible shortcomings provides a path for continuing to improve model inference through larger, higher-quality data sets.

#### Powerful Combined Approach Provides Effective Strategy to Obtain Bioactivation Kinetics.

Kinetic assessments are valuable in determining the mechanisms and efficiencies of reactive metabolite formations that often correlate with toxicological outcomes (Thompson et al., 2016). Although a common approach, mass spectrometry necessitates the use of costly and often difficult-to-synthesize authentic standards because of varied responses among substrates and metabolites but it is exceptional for characterizing metabolite and adduct structures to validate bioactivations (Scalbert et al., 2009; Dahal et al., 2011; Aretz and Meierhofer, 2016). Consequently, we used this technique to infer the structure of three putative reactive quinones from four observed adducts based on fluorescence. The latter adduct could not be characterized using mass spectrometry. This outcome may reflect the limit of detection, poor ionizability, or multiple glutathione adduction of a reactive metabolite. The possibility of more than one dansylated glutathione reacting with reactive quinones would lead to much higher signals than the single adducts. This outcome was previously observed by Kang et al., (2009) in the case of the structurally similar compound lumiracoxib, and so that may be the case for the observed meclofenamate metabolite in this study. Despite these insights from mass spectrometry, the approach cannot provide quantitative information on yields of adducts in the absence of authentic standards for meclofenamate quinone metabolites. The fluorescent labeling approach developed by Gan et al. (2005) provides a powerful solution to infer quantitation of reactive quinones trapped with glutathione. We report the first evidence that variations in structure among dansylated molecules do not alter the fluorescence response. Moreover, fluorescence responses in our study did not correlate with mass-spectroscopic responses, adding further evidence to the unreliability of mass spectrometry-based quantitation of analytes without standards. We observed that the ratio of mass-spectroscopic and fluorescent responses varied between 10- and 100-fold, indicating that mass-spectroscopic and fluorescent responses are not correlative nor are mass-spectroscopic responses useful to quantify analytes without the use of an authentic standard. Taken together, dansyl-labeled glutathione adducts in our study provided a way to accurately quantify without the need of authentic standards, and, importantly, our control studies validated this robust approach as a practical, generalizable strategy for quantifying any reactive metabolites trapped by dansylated reagents.

#### Meclofenamate Quinones Are Formed through Multiple Kinetics Mechanisms.

Meclofenamate bioactivation involves at least two oxidative steps to generate a reactive quinone that undergoes subsequent trapping by glutathione. Despite that complexity, bioactivation kinetics may conform to a simple Michaelis-Menten mechanism when a single step dominates the kinetics for the pathway. The metabolism of meclofenamate to the monohydroxy para-quinone-imine or the suspected multiply glutathionylated metabolite reflected that outcome. Nevertheless, the kinetic profiles for the dechloro-ortho-quinone-imine and the dihydroxy ortho-quinone demonstrated positive cooperativity in which a low efficient bioactivation reaction transitions to a more efficient one. A possible explanation for these mechanisms could be reaction steps that are poorly coupled at low substrate concentrations yet improve at higher concentrations. Typically, this outcome occurs when the second reaction step is a low-affinity reaction so that intermediates accumulate until saturation of the second reaction step, resulting in coupled reactions. Alternatively, these kinetics could be indicative of multiple-substrate binding to a single enzyme, whereby binding at an initial site leads to more positive (cooperative) binding interactions for the second event. Although both mechanisms are possible, the latter cooperative one seems likely given the kinetics reported for the similarly structured NSAID diclofenac. CYP2C9 and CYP3A4 metabolism of diclofenac involves multiple-substrate binding during turnover (Zhang et al., 2004; Kumar et al., 2006; Denisov et al., 2009; Řemínek and Glatz, 2010). As a follow-up, future studies could focus on identifying possible cytochromes P450 responsible for these reactions and exploring possible mechanisms for meclofenamate bioactivation.

Among all four bioactivation reactions (Supplemental Fig. 5), the most efficient was that for the monohydroxy para-quinone-imine followed by the dechloro-ortho-quinone-imine. The pathways leading to the dihydroxy ortho-quinone and suspected multiply glutathionylated metabolite were very inefficient. These observations are chemically plausible given that formation of the monohydroxylated para-quinone-imine is more energetically favored than formation of either the dechloro-ortho-quinone-imine or dihydroxy ortho-quinone, as it requires one fewer metabolic step to form. The dechlorinated ortho-quinone-imine can only form after enzymatic removal of one chlorine atom, which requires an additional catalytic step. Finally, both the dihydroxylated ortho-quinone and the suspected multiply glutathionylated metabolite formed with low efficiencies because of the complex and numerous metabolic steps required to generate them. Consequently, the monohydroxy para-quinone-imine followed by the dechloro-ortho-quinone-imine are the dominant bioactivation pathways for meclofenamate, yet the relevance of those pathways will depend on variations in cytochrome P450 levels responsible for those reactions in the general population.

#### Meclofenamate Bioactivation Pathways May Provide Cause for Possible Hepatoxicity.

Knowledge of meclofenamate bioactivation provides insights on the possible cause for toxic risks. Meclofenamate is labeled as “ambiguous DILI concern” in the Food and Drug Administration DILIrank database, which is a comprehensive database of known hepatotoxicant drugs marketed or previously marketed in the United States. This classification indicates data are available on hepatotoxicity frequency, severity, and causality for meclofenamate, yet there is insufficient information for a definitive ranking on the severity of its DILI risk. Importantly, no prior reported studies exist on the mechanisms by which meclofenamate may induce hepatotoxicity. We have shown that meclofenamate is highly bioactivated into reactive quinone metabolites, which may deplete hepatocellular ATP and induce changes in the mitochondrial permeability transition, thereby causing hepatoxicity, as previously discussed by Masubuchi et al. (1999) and Li et al. (2009). Suppression of those deleterious effects occurs when proteins react with reactive meclofenamate metabolites to form adducts, including those involving glutathione. Nevertheless, protein modifications can lead to their dysfunction in biologic processes and/or form antigens that elicit immune-mediated liver toxicity (Goldkind and Laine, 2006; Sriuttha et al., 2018). The formation of quinone-species metabolites as a precursor to hepatotoxicity has been extensively studied for many different drugs (Fan and Bolton, 2001; Ishihara and Shimamoto, 2008; Ishihara et al., 2011; Ishihara, 2013; Bolton and Dunlap, 2017; Hughes and Swamidass, 2017), and it is reasonable that the quinone-species metabolites observed in this study may also attribute to hepatotoxic events caused by meclofenamate dosage. In this study, calculated fractional bioactivation values based on kinetics provide a tractable strategy for assessing reactive metabolite loads from individual and combined bioactivation pathways to toxicological and clinical outcomes. Unfortunately, quality data are lacking on sufficient clinical outcomes to explore the potential importance of reactive metabolite burden on toxicity for meclofenamate.

## Concluding Remarks

Herein, we demonstrated that computational models predicted many possible meclofenamate bioactivations and metabolites, but establishing relevant pathways required novel and quantitative kinetic experiments. Computational modeling rapidly yielded predictions for metabolism and bioactivation without limitations on the feasibility of experimental investigations. The reliability of model predictions may not be clear, yet outputs were useful in facilitating analysis of experimental data. Gaps in correlations indicated areas in which models can be improved to better realize the potential in improving the efficiency of these types of studies. Moreover, our experimental studies demonstrated the first practical application of dansyl glutathione to directly quantify adducts based off fluorescent response and assess bioactivation kinetics. Steady-state kinetics studies revealed which bioactivations occur, their fractional contributions to overall metabolism, and hence their potential to cause toxicity. The combined modeling strategy in this study facilitated the prediction of reactive metabolite structures, whereas data generated from this study provided a tractable strategy to assess reactive metabolite load more accurately for future comparative studies with other NSAIDs and drugs in general. In fact, several well used and structurally similar drugs are marketed on or withdrawn from the United States market that may reflect contributions from bioactivation pathways (Kang et al., 2009; Pillans et al., 2012). Our current studies show a combination of robust computational and experimental approaches that could be expanded to identify quantitative structure-function relationships determining drug bioactivation potential and yield more accurate assessments for attempting to correlate those findings to toxicological outcomes.

## Abbreviations

DILI: drug-induced liver injury
f_ba_: fractional bioactivation
GSH: glutathione
HLM150: human liver microsomes 150
NSAID: nonsteroidal anti-inflammatory drug
